# Risk factors for depression and anxiety in pregnant women during the COVID-19 pandemic: Evidence from meta-analysis

**DOI:** 10.1371/journal.pone.0265021

**Published:** 2022-03-04

**Authors:** Yupeng Luo, Kui Zhang, Mengxue Huang, Changjian Qiu

**Affiliations:** 1 West China hospital, Sichuan University, Chengdu, Sichuan, People’s Republic of China; 2 Department of Forensic Pathology, West China School of BasicMedical Sciences & Forensic Medicine, Sichuan University, Chengdu, Sichuan, People’s Republic of China; 3 Hua Da appraisal center, Chengdu, Sichuan, People’s Republic of China; Babol University of Medical Science, ISLAMIC REPUBLIC OF IRAN

## Abstract

**Background:**

The prevalence of anxiety and depression in pregnant women has significantly increased after the spread of COVID-19 throughout the world. We carried out this meta-analysis to reveal the information about risk factors for depression and anxiety in pregnant women during the COVID-19 pandemic.

**Methods:**

We searched the PubMed, Embase and CNKI (China National Knowledge Infrastructure) databases for all articles. The odds ratio (OR) corresponding to the 95% confidence interval (95% CI) was used to assess the risk factors for mental health. The statistical heterogeneity among studies was assessed with the Q-test and *I*^*2*^ statistics.

**Results:**

We collected 17 studies including 15,050 pregnant women during the COVID-19 pandemic. Our results found that factors including decrease in the perception of general support and difficulties in household finances have damage effects on anxiety, and factors including undereducated, unemployed during pregnancy, with a chronic physical illness before pregnancy, decrease in the perception of general support, difficulties in household finances, disobey the isolation rules, and smoking during pregnancy have increased risk of depression.

**Conclusion:**

Our meta-analysis revealed some risk factors for mental health in pregnant women during COVID-19 pandemic. Mental health interventions in pregnant women may involve targeted methods individually.

## Introduction

COVID-19 has rapidly spread throughout the world, with the total number of cases exceeding 238 million and resulting in more than 4.8 million deaths globally as of October 12, 2021. The COVID-19 pandemic has drastically changed the daily lives worldwide [1]. Although researches have revealed that there is currently no evidence of vertical transmission in women who develop COVID-19 pneumonia in pregnancy [2–4], many pregnant women still worry about going to hospitals because of the fear of COVID-19 infection [5, 6].

Prenatal mental health in pregnant women is a worldwide public health issue and affect up to 20% of women during pregnancy and the postpartum period [7]. Pregnant women are more likely to develop anxiety and depression during the COVID-19 pandemic. Changes in women’s hormone levels may lead to an increased chance of depression progression twice that of men, especially during the reproductive period and pregnancy [8]. Thus, women are more likely to experience anxiety and depression symptoms during COVID-19 than men [9, 10]. The prevalence of anxiety in pregnant women has been reported to range from 26% to 57%, the overall prevalence of depression has been reported to range from 20% to 31% [11]. Prenatal mental health pose heavy burden not only for pregnant women themselves but also for their offspring [12]. Accumulated evidence shown that prenatal psychological problems adversely affect the babies. Stress related anxiety during pregnancy may result in fetal death or fetal abnormalities [13]. Furthermore, the offspring of mothers who experience psychological distress during pregnancy are more likely to have cognitive and behavioral problems and their communication skills are significantly affected [14–16].

As mentioned above, the prevalence of anxiety and depression in pregnant women has significantly increased after the spread of COVID-19 throughout the world, and that may substantially pose adverse effect on the offspring. However, there is no definitive information about risk factors for depression and anxiety in pregnant women during the COVID-19 pandemic. In the present study, we carried out this meta-analysis to fill this void. This study was reported in accordance with the PRISMA statement for reporting systematic reviews and meta-analysis [17].

## Methods

### Publication search and inclusion criteria

**Fig 1:**
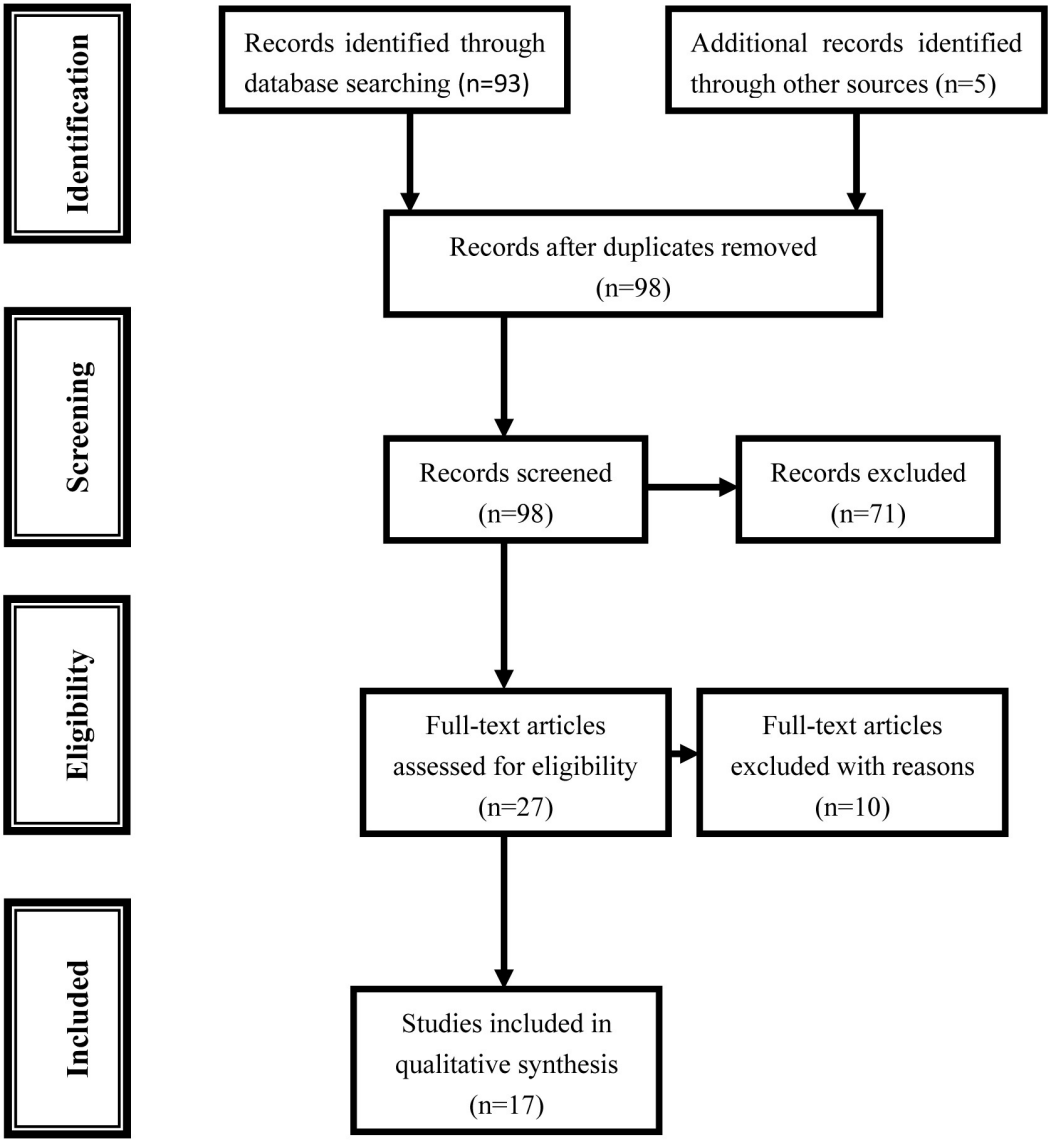
Flowchart for identification of studies.

We searched the PubMed, Embase and CNKI (China National Knowledge Infrastructure) databases for all articles within a range of published years from 2019 to 2021 on risk factors for depression and anxiety in pregnant women during the COVID-19 pandemic (last search was August 16, 2021). The following terms were used in this search: ‘pregnant’, ‘mental’, ‘anxiety’, ‘depression’ and ‘COVID-19’. Please refer to S1 File for the electronic search strategy. In order to identify the relevant publications, the references cited in the research papers were also scanned. Combining searches resulted in 93 abstracts. In addition, five studies were identified through review articles and meta-analysis, for a total of 98 studies were screened after duplicated records were removed. After screening the titles and abstracts, 26 were retrieved for more detailed evaluation (Fig 1). We used the Newcastle-Ottawa Scale (NOS) for assessing the quality of cohort studies and case-control studies based on three categories and eight items.

We evaluated the eligible studies if all the following conditions were met: (1) evaluation on risk factors for depression and anxiety in pregnant women during the COVID-19 pandemic; (2) inclusion of sufficient data or the data can be acquired from the manuscript or supplementary materials to calculate ORs and 95% CIs; (3) the diagnosis of depression or anxiety with qualified criteria; and (4) the study was published in English.

### Data extraction

Two authors (Yupeng Luo and Kui Zhang) independently reviewed and extracted the data needed. Disagreements were resolved through discussion among the authors to achieve a consensus. The following information was recorded for each study: first author, year of publication, region, risk factors, diagnostic criteria, cases and population (all of the data are shown in Table 1).

**Table 1:** Characteristics of literatures included in the meta-analysis.

Reference	Year	Region	Anxiety				Depression			
			Risk factors	Diagnostic criteria	Cases	Population	Risk factors	Diagnostic criteria	Cases	Population
Hamzehgardeshi Z [36]	2021	Iran	Parity, chronic illness	CDA-Q	67	318				
Kahyaoglu Sut H [1]	2021	Turkey	Education, working status during pregnancy, chronic illness, regular physical activity, follow the isolation rules	HADS- anxiety scores≥8	260	403	Education, working status during pregnancy, chronic illness, regular physical activity, smoking during pregnancy, follow the isolation rules	HADS- depression scores≥8	227	403
Shangguan F [12]	2021	China	Age, education, chronic illness, general support, family annual income	GAD-7 scale≥5	459	2,120				
Lebel C [23]	2021	Canada	Working status during pregnancy, regular physical activity, general support, follow the isolation rules	PROMIS Anxiety T-scores		1,983	Working status during pregnancy, regular physical activity, follow the isolation rules, general support	EPDS		1,983
Matsushima M [24]	2020	Japan	Family annual income, general support	EPDS ≥13	302	1,777	Family annual income, general support, working status during pregnancy	EPDS ≥13	302	1,777
Nowacka U [25]	2021	Poland	Working status during pregnancy, follow the isolation rules	GAD-7 scale ≥6	165	439				
Thayer ZM [26]	2021	USA					Age, education, parity, chronic illness, family annual income	EPDS	504	2,099
Koyucu RG [27]	2021	Turkey	Age, parity, working status during pregnancy, chronic illness, general support	DASS	453	729	Age, parity, working status during pregnancy, chronic illness, family annual income, general support	DASS	325	729
Maharlouei N [8]	2021	Iran	Age, education, parity, working status during pregnancy, chronic illness, family annual income	DASS-21	105	540	Age, education, parity, working status during pregnancy, chronic illness, family annual income	DASS-21	28	540
Mappa I [28]	2020	Italy	Education, parity, working status during pregnancy	STAI-T	68	178				
Mappa I [28]	2020	Italy	Education, parity, working status during pregnancy	STAI-S	137	178				
Durankus F [29]	2020	Turkey					Parity, working status during pregnancy	EPDS ≥13	92	260
Ding W [30]	2021	China	Age, education, parity, working status during pregnancy, family annual income	SAS	170	817				
Jiang H [31]	2020	China	Age, education, parity, working status during pregnancy	SAS	339	1,873	Age, education, parity, working status during pregnancy	EPDS	859	1,873
Ceulemans M [32]	2021	Europe					Chronic illness, smoking during pregnancy	EPDS	586	3,907
Wu F [33]	2021	China	Age, education, parity, working status during pregnancy, regular physical activity, follow the isolation rules	GAD-7	337	3,434	Age, education, parity, working status during pregnancy, regular physical activity, smoking during pregnancy, follow the isolation rules	PHQ-9	228	3,434
Patabendige M [34]	2020	Sri Lanka	Age, education, parity, working status during pregnancy, family annual income	HADS	45	257	Age, education, parity, working status during pregnancy, family annual income	HADS	50	257
Nurrizka RH [35]	2021	Indonesia	Age, education, working status during pregnancy	DASS-21	64	120				

CDA-Q, the Corona Disease Anxiety Questionnaire; HADS, hospital anxiety and depression scale; GAD-7, generalized anxiety disorder-7; EPDS, edinburgh postnatal depression scale; DASS, depression anxiety stress scale; DASS-21, the short form of the depression anxiety stress scales; STAI-T, the State–trait anxiety inventory validated test for scoring trait anxiety; STAI-S, the State–trait anxiety inventory validated test for scoring state anxiety; SAS, Self-Rating Anxiety Scale; PHQ-9, patient health questionnaire 9.

### Statistical analysis

The odds ratio (OR) corresponding to the 95% confidence interval (95% CI) was used to assess the risk factors for depression and anxiety in pregnant women during the COVID-19 pandemic. The statistical heterogeneity among studies was assessed with the Q-test and *I*^*2*^ statistics [18]. If there was no obvious heterogeneity, the fixed-effects model (the Mantel-Haenszel method) was used to estimate the summary OR [19]; otherwise, the random-effects model (the DerSimonian and Laird method) was used [20]. Finally, random effects models were used to calculate the overall OR estimates and 95% CIs to assess the risk factors for depression and anxiety in pregnant women during the COVID-19 pandemic. To explore sources of heterogeneity across studies, we did logistic meta-regression analyses. We examined the following study characteristics: publication year, region, number of cases, and number of population. Publication bias was evaluated with funnel plot and Begg’s rank correlation method [21]. The statistical analyses were performed by STATA 12.0 software (Stata Corp., College Station, TX).

## Results

### Characteristics of studies

Out of a total of 98 titles and abstracts, 26 were retrieved for more detail evaluation. Of the ten excluded studies, two papers were reviews, seven papers lacked enough data, and one paper was excluded with duplicated data [22] and the updated data were included [23]. Finally, 17 studies [1, 8, 12, 23–36] met the inclusion criteria for this study, including 15,050 pregnant women during the COVID-19 pandemic. The details in selected studies were listed in Table 1.

### Quantitative synthesis

**Fig 2:**
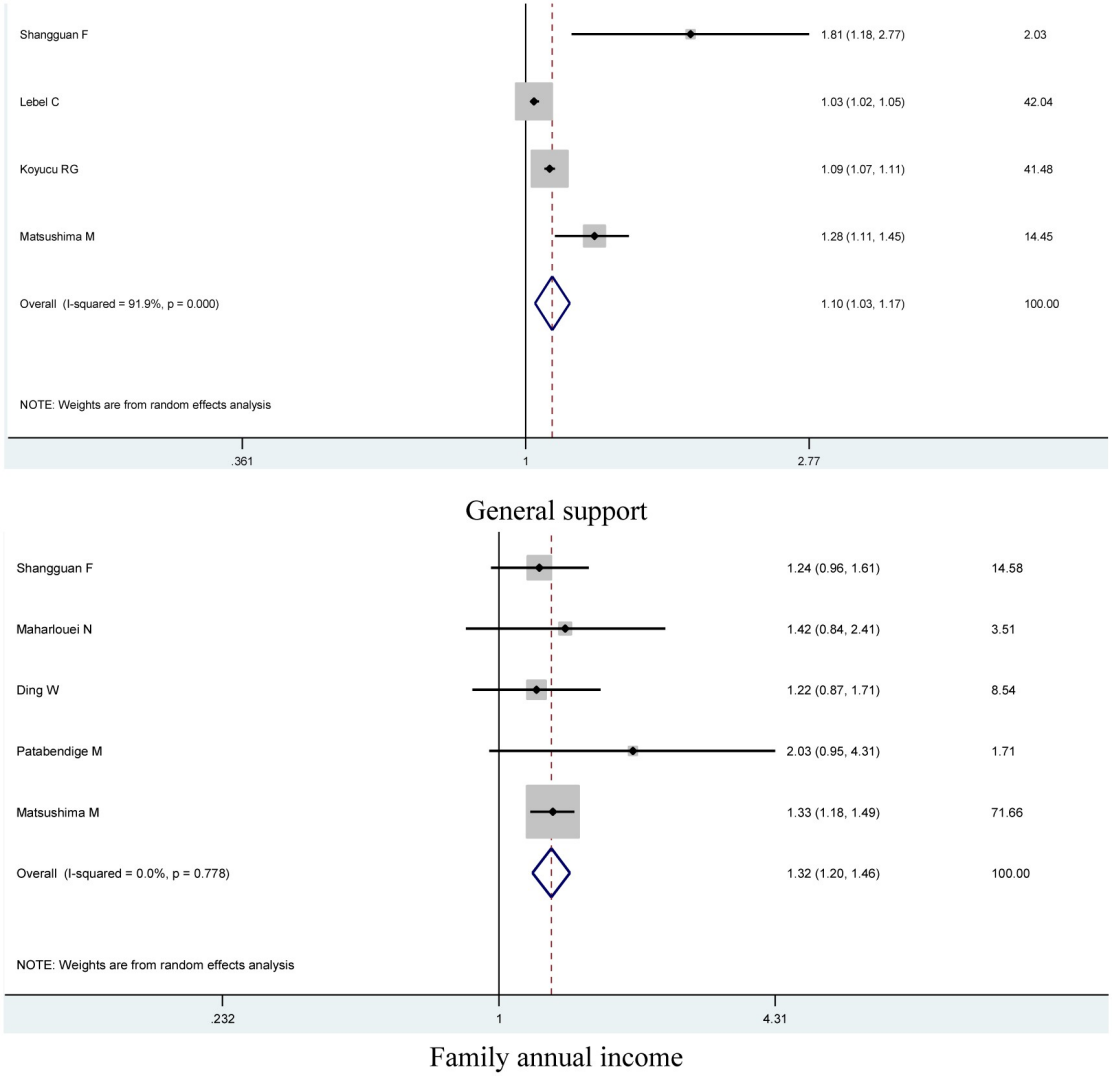
Forest plot of risk factors and anxiety in pregnant women.

For anxiety, factors including age, education, parity, working status during pregnancy, chronic illness, regular physical activity, general support, family annual income, and follow the isolation rules were assessed in pregnant women during the COVID-19 pandemic. Finally, decrease in the perception of general support, smoking during pregnancy and difficulties in household finances have damage effects on anxiety during pregnancy amid the COVID-19 pandemic (OR = 1.10, 95% CI = 1.03–1.17 for decrease in the perception of general support, OR = 3.00, 95% CI = 1.77–5.09 for smoking during pregnancy, and OR = 1.32, 95% CI = 1.20–1.46 difficulties in household finances, shown in Table 2 and Fig 2).

**Table 2:** Associations between risk factors and anxiety or depression in pregnant women.

	Anxiety				Depression			
	N^a^	Case/control	OR (95%CI)	*P* ^b^	N^a^	Case/control	OR (95%CI)	*P* ^b^
Age	8	1,972/9,890	1.01(0.96–1.06)	0.160	6	1,994/8,932	1.00(0.94–1.07)	0.024
Education	10	1,984/9,920	1.15(0.86–1.54)	<0.001	6	1,896/8,606	**1.41(1.10–1.81)**	0.050
Parity	9	1,721/8,324	0.97(0.78–1.19)	0.039	7	2,086/9,192	0.94(0.76–1.17)	0.010
Working status during pregnancy	13	2,445/12,728	1.17(0.89–1.53)	<0.001	9	2,111/11,256	**1.68(1.25–2.25)**	0.017
Chronic illness	5	1,344/4,110	1.84(0.80–4.22)	<0.001	5	1,670/7,678	**2.10(1.13–3.90)**	<0.001
Regular physical activity	3	597/5,820	1.09(0.89–1.33)	0.107	3	455/5,820	1.51(0.91–2.53)	<0.001
General support	4	1, 214/6,609	**1.10(1.03–1.17)**	<0.001	3	627/4,489	**1.06(1.03–1.10)**	0.034
Family annual income	5	1,081/5,511	**1.32(1.20–1.46)**	0.778	5	1,209/5,402	**1.76(1.24–2.50)**	0.001
Follow the isolation rules	4	729/6,259	1.47(0.88–2.43)	<0.001	3	455/5,820	**1.05(1.05–1.05)**	0.782
Smoking during pregnancy	2	597/3837	**3.00(1.77–5.09)**	<0.001	3	1,041/7,744	**2.91(2.04–4.16)**	0.396

^a^ Number of comparisons.

^b^ P value of Q-test for heterogeneity test.

Boldfaced values indicate a significant difference at the 5% level.

**Fig 3:**
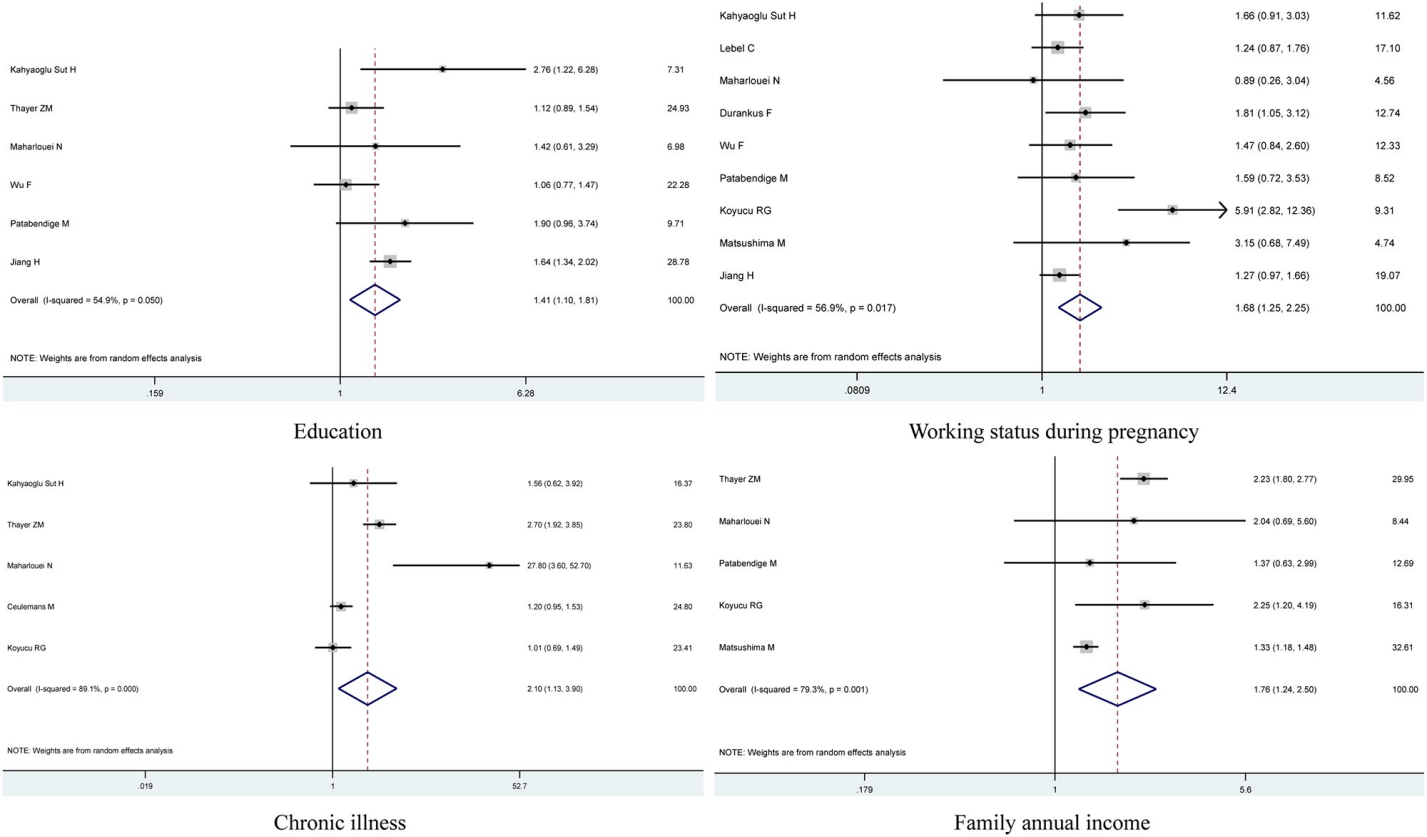
Forest plot of risk factors and depression in pregnant women.

For depression, factors including age, education, parity, working status during pregnancy, chronic illness, regular physical activity, general support, family annual income, smoking during pregnancy, and follow the isolation rules were assessed in pregnant women during the COVID-19 pandemic. Finally, undereducated, unemployed during pregnancy, with a chronic physical illness before pregnancy, decrease in the perception of general support, difficulties in household finances, disobey the isolation rules, and smoking during pregnancy have increased risk of depression during pregnancy amid the COVID-19 pandemic (OR = 1.41, 95% CI = 1.10–1.81 for undereducated, OR = 1.68, 95% CI = 1.25–2.25 for unemployed during pregnancy, OR = 2.10, 95% CI = 1.13–3.90 for chronic physical illness before pregnancy, OR = 1.06, 95% CI = 1.03–1.10 for decrease in the perception of general support, OR = 1.76, 95% CI = 1.24–2.50 for difficulties in household finances, OR = 1.05, 95% CI = 1.05–1.05 for disobey the isolation rules, and OR = 2.91, 95% CI = 2.04–4.16 for smoking during pregnancy, shown in Table 2 and Fig 3).

### Evaluation of heterogeneity

To explore sources of heterogeneity across studies, we did logistic meta-regression analyses. Logistic meta-regression analyses found no possible factors that may substantially influence the initial heterogeneity.

### Sensitivity analysis

The influence of a single study on the overall meta-analysis estimate was investigated by omitting one study at a time, and the omission of any study made no significant difference, indicating that our results were statistically reliable.

### Publication bias

The Begg’s test was performed to evaluate the publication bias of selected literatures. No evidence of publication bias in our study was observed (all *P* > 0.05).

## Discussion

The prevalence of anxiety and depression among pregnant women increased significantly during the COVID-19 epidemic. Our meta-analysis found that facors including decrease in the perception of general support, smoking during pregnancy and difficulties in household finances have damage effects on anxiety during pregnancy, and factors including undereducated, unemployed during pregnancy, with a chronic physical illness before pregnancy, decrease in the perception of general support, difficulties in household finances, disobey the isolation rules, and smoking during pregnancy have increased risk of depression during pregnancy amid the COVID-19 pandemic.

Education is an important factor related to the development of anxiety and depression during pregnancy. Although our results only found higher risk of depression in pregnant women with low education levels, pregnant women with low education levels have been reported to be a high risk of developing both anxiety and depressive symptoms [8, 37]. This may be explained by the fact that pregnant mothers with a high level of education had more awareness and were easier to access the correct information of COVID-19 pandemic than low-educated pregnant mothers [8, 38], and were better adapted to pandemic conditions [39].

In accordance with previous findings [40, 41], the present study revealed that the risk of depression is higher in pregnant women who are not working during the pandemic. Nanjundaswamy et al. [42]found that approximately 35% of pregnant women in India have job related concerns. Being unemployed or being a housewife during the pandemic increases the time spent at home and reduces socialization and interpersonal communication, thereby may increase the risk of anxiety and depression.

Physical activity may play an important role in the management of mild-to-moderate mental health diseases, especially depression and anxiety [43]. WHO 2020 guidelines on physical activity and sedentary behavior [44] provides the recommendation for regular strength training to be included for pregnant women. Previous studies indicated that regular activity during pregnancy can reduce the likelihood of anxiety and depression [11, 41]. Our research with limited data failed to confirm the above result.

Chronic illness have been stressed as high risk for complications in severe COVID-19 patients with increased disease severity and mortality [45–47], therefore, pregnant women with a history of chronic illness may be more anxious than those without. As a result of psychological distress, pregnant women may choose not to receive antenatal care at health facilities due to worries about being infected with COVID-19 [48]. Our research found increased risk of depression in pregnant women with a chronic physical illness before pregnancy.

Decrease in the perception of general support and difficulties in household finances were important factors associated with both anxiety and depression during pregnancy. lack of social and or partner support and or family care is closely associated with increased risk of prenatal symptoms of anxiety and depression [49, 50], prenatal anxiety was related to nobody providing support in everyday life [12]. Prior research has found that lower income and financial struggles are associated with increased risk of poor mental health in pregnancy [51]. During the COVID-19 pandemic these financial stressors have only increased, with record unemployment. Finally, financial stress was significantly associated with the likelihood of having clinically significant anxiety and depression during COVID-19 pandemic.

Self-isolate at home may make the pregnant women feel secure during COVID-19 pandemic, but spending more time with their intimate partners may also increase partner violence, especially emotional abuse, which can lead to unhealthy emotions and even adverse birth outcomes for pregnant women [37]. Previous study have shown that social distancing and isolation at home after the COVID-19 pandemic has greatly impacted human health, causing sudden lifestyle changes with accompanying social and economic consequences [52].

Interestingly, we also found that pregnant women who smoked were at higher risk of depression and anxiety. Recent study suggested that depression appears to be associated with smoking dependence and mediated by neuroticism [53]. Otherwise, attempting to maintain better mood may be a motivating factor for smoking among depressed individuals [54]. Furthermore, pregnancy is a stressful event that alters women’s hormonal balance [55], and thus pregnant women might tend to respond to their uncomfortable feelings by smoking and drinking.

A few limitations of our study should be considered. There was heterogeneity among studies although we performed logistic meta-regression analyses and stratified analysis to explore sources of heterogeneity across studies, we still found no possible factors that may substantially influence the initial heterogeneity, and the heterogeneity may potentially affect the results. Moreover, although we did not observe significant publication bias, publication bias is possible in any meta-analysis.

In conclusion, our meta-analysis indicated that education status, unemployed during pregnancy, with a chronic physical illness before pregnancy, general support, household finances, disobey the isolation rules, and smoking during pregnancy were risk factors for mental health in pregnant women during COVID-19 pandemic. Mental health interventions in pregnant women may involve targeted methods individually.

### Relevance for clinical practice

The present meta-analysis found that factors including decrease in the perception of general support and difficulties in household finances have damage effects on anxiety, and factors including undereducated, unemployed during pregnancy, with a chronic physical illness before pregnancy, decrease in the perception of general support, difficulties in household finances, disobey the isolation rules, and smoking during pregnancy have increased risk of depression in pregnant women during the COVID-19 pandemic. The prevalence of anxiety and depression in pregnant women has significantly increased after the spread of COVID-19 throughout the world, and that may substantially pose adverse effect on the offspring. Our meta-analysis revealed some risk factors for mental health in pregnant women, and provided advices that mental health interventions in pregnant women during COVID-19 pandemic may involve targeted methods individually.

## Supporting information

S1 File Electronic search strategy. (DOCX)

S1 Checklist (DOCX)
