# Supplementary material for: Risk factors for depression and anxiety in pregnant women during the COVID-19 pandemic: Evidence from meta-analysis
Source: PLoS One. 2022 Mar 4;17(3):e0265021. doi: 10.1371/journal.pone.0265021 (PMC8896698; doi:10.1371/journal.pone.0265021)
Supplement: S1 File — (DOCX) [file pone.0265021.s001.docx]

S1 File. Electronic search strategy

Library/Database: PubMed

Date of Search: August 16, 2021

Search String: ("COVID-19") AND ("pregnant”) AND("mental” OR " anxiety” OR " depression”)

# Hits: 93

Library/Database: Embase

Date of Search: August 16, 2021

Search String: ("COVID-19") AND ("pregnant”) AND("mental” OR " anxiety” OR " depression”)

# Hits: 89

Library/Database: CNKI

Date of Search: August 16, 2021

Search String: ("COVID-19") AND ("pregnant”) AND("mental” OR " anxiety” OR " depression”)

# Hits: 5
